# Pattern of congenital heart disease among Egyptian children: a 3-year retrospective study

**DOI:** 10.1186/s43044-021-00133-0

**Published:** 2021-01-29

**Authors:** Marwa Moustapha Al-Fahham, Yasmin Abdelrazek Ali

**Affiliations:** 1 Pediatric Department, Faculty of Medicine, Ain Shams University, Cairo, Egypt; 2 Al-Salam International Hospital/Doctor Residency Building, P.O. Box 11023, Bneid Al Gar, Kuwait; 3 Cardiology Department, Faculty of Medicine, Ain Shams University, Cairo, Egypt

**Keywords:** Congenital heart defects, Perinatal risk factors, Consanguinity, Maternal illnesses, Maternal age

## Abstract

**Background:**

Congenital heart disease (CHD) is a multifactorial birth defect which has variable demographic characteristics among children in different geographical areas. This study aimed to detect the distribution of demographic data, perinatal risk factors, types, age, and mode of presentation of CHD among Egyptian children.

**Results:**

The medical records of 1005 patients were included. They were 545 males (54%) and 462 females (46%) with a ratio of 1.2:1. Acyanotic CHD was encountered in 79.2%. Isolated ventricular septal defect and tetralogy of Fallot were the most common acyanotic and cyanotic lesions, respectively. The majority was diagnosed within the first year of life (86.7%) and was born to young mothers (91.3%). The accidental discovery of a murmur was the most frequent presentation (35%). Heart failure was detected in 44%, audible murmurs in 74.4%, maternal illnesses in 54%, consanguinity in 44.6%, prematurity in 19.3%, assisted reproduction in 11.7%, family history of CHD in 9.2%, abortions in 7.1%, and extracardiac anomalies in 3.6% of the studied population. Down syndrome (DS) was the most commonly occurring chromosomal anomaly, and the atrioventricular septal defect was the most characteristic cardiac lesion found among them.

**Conclusions:**

There is no sex predilection among Egyptian children with CHD. Most of the cases are diagnosed in early infancy. Accidental discovery of a murmur is the most common mode of presentation. A variety of predisposing risk factors are abundant in the Egyptian population. DS is the most common chromosomal anomaly linked to CHD. Establishment of a national medical birth registry containing all information about all births in Egypt is needed for adequate surveillance and monitoring of perinatal health problems and congenital birth defects so that preventive measures can be early implemented. Proper and detailed data collection should be fulfilled in the medical records of every single patient.

## Highlights

- The majority of patients (79.2%) had acyanotic CHD.
- VSD is the most commonly encountered acyanotic CHD.
- Fallot’s tetralogy is the most commonly occurring cyanotic CHD.
- No sex predilection among the studied population.
- Consanguinity is prevalent in 44.6% of the studied population.

## Background

Congenital heart disease (CHD) is one of the relatively common congenital disabilities whose prevalence ranges from 3.5-17.5 per 1000 live births [1]. They are becoming an increasing cause of pediatric mortality [2], especially in the developing countries [3]. The clinical spectrum of CHD is versatile and changes according to the age of presentation. Asymptomatic presentation is common and discovered accidentally on routine checkup visits, whereas other presentations can range from poor suckling, cyanosis, and shortness of breath up to frank heart failure [4].

Most of these defects follow the multifactorial pattern of inheritance as a result of interlinking genetic and environmental factors with a smaller percentage being linked to chromosomal aberrations [5]. The pattern of risk factors for CHD is different among different parts of the world. In developing countries, consanguinity is relatively prevalent; most mothers are housewives, non-smokers, and non-alcohol consumers [6]. Unfortunately, only a few studies had evaluated the perinatal risk factors among those populations [7].

A meticulous study of the epidemiology of CHDs is the cornerstone for better identification of the etiology for cardiac dysmorphogenesis so that every opportunity can be offered to prevent them prenatally [8–10]. Unfortunately, the epidemiology of CHD had not been thoroughly studied among Egyptian children; hence, this study aimed to review the risk factor portfolio, relative frequencies of each type of CHD, demographic characteristics, age, and mode of clinical presentations among Egyptian children with CHD; so that appropriate changes in preventive health policies can be implemented and optimum care for such patients can be provided.

## Methods

This is a retrospective epidemiological cross-sectional study that included all children with confirmed CHDs who are registered in the Pediatric Cardiology Clinic at a Children’s University Hospital which is considered a tertiary referral center that provides medical services for a large geographical sector in Cairo Governorate (mainly urban areas). Collaboration between all departments (pediatrics, cardiology, fetal ultrasound unit, and cardiothoracic surgery) takes place in this university hospital to provide all the necessary facilities needed for proper diagnosis and management of children with CHD (including advanced pediatric echocardiography, cardiac catheterization, fetal echocardiography, and cardiothoracic surgeries).

The study was conducted over a period of 3 years from January 2013 till December 2015. It included all children with confirmed structural CHD who were diagnosed from the first day of life up till the age of 12 years. Patients with congenital cardiomyopathies and congenital heart block were excluded from the study.

The files of 1005 patients were revised in detail. Some missing data were detected in 57 files and were completed by telephone calls when a valid number was available. Diagnosis of the type of CHDs was confirmed by echocardiography and documented in the files. Data collected included full demographic and clinical data. A thorough study of the perinatal (antenatal, natal, and postnatal), as well as the family history, was done to detect possible underlying risk factors for CHDs.

Demographic data included age at diagnosis, gender, sex, consanguinity, parental education, and occupation. For each studied case, the presenting complaint, type of CHD, presence of dysmorphic features, associated syndromes, or other congenital anomalies were all reported. Complete risk factor assessment was done for each reviewed file. Antenatal history included maternal age at conception, full obstetric history, repeated abortions or stillbirths, type of conception (normal or assisted), medical diseases with pregnancy (i.e., diabetes mellitus, hypertension, systemic lupus, and bronchial asthma), teratogen exposure (radiation, chemicals and smoking), maternal infection (i.e., urinary tract infection, congenital TORCH infections, and premature rupture of membranes), maternal medications during pregnancy (i.e., hypoglycemic, antihypertensive, antiepileptic, antibiotics). Natal and postnatal history included the gestational age (GA), early neonatal illnesses, and NICU admissions. Family history included the presence of CHDs, other congenital or chromosomal abnormalities, and sibling deaths.

### Statistical methods

Data were collected, tabulated, and analyzed using SPSS, version 12. Mean and standard deviation were used for quantitative variables. Frequency and percentages were used for qualitative variables.

## Results

Over the 3 years study period, a total number of 1005 of patients were included. They were 543 males (54%) and 462 females (46 %), their ages at diagnosis ranged from 1 day to 12 years with a median and interquartile range (IQR) of 6 (9-0.5) months. Of the studied population, acyanotic CHD was encountered in 796 (79.2%) whereas cyanotic CHD in 209 (20.8%). The isolated ventricular septal defect was the most common acyanotic CHD (19.8%), while tetralogy of Fallot (9.8%) was the most common cyanotic CHD. Small hemodynamically insignificant patent foramen ovale (PFO) and patent ductus arterisus (PDAs) were considered normal. Most of our patients had been diagnosed within the first year of life (48.9% in the early infancy and 37.8% in the neonatal period). The relative frequencies and ages of presentations of individual types of CHDs are shown in Table 1.

**Table 1:** Frequency and age-wise distribution of types of CHD

Age group	Neonatal	> 1 month-1 year	> 1-< 5 years	5-12 years	Total	Percentage
Isolated VSD	60	105	28	6	199	19.8
Isolated ASD	54	66	31	2	153	15.2
Tetralogy of Fallot	45	39	12	3	99	9.8
AVSD	40	50			90	8.9
Pulmonary stenosis^a^	10	54	11	8	83	8.3
ASD/VSD	11	62			73	7.3
ASD/PDA	14	42	1	1	58	5.8
Complex CHD	40	17			57	5.7
VSD/PDA	15	24			39	3.9
TGA	31				31	3.1
Mitral valve prolapse^a^			6	24	30	3
Coarctation of aorta	18	10			28	2.8
Tricuspid atresia^a^	15				15	1.5
Hypoplastic left heart	4	10			14	1.4
TAPVR		13			13	1.3
Pulmonary atresia	13				13	1.3
Ebstein anomaly	10				10	1
Total	380	492	89	44	1005	
Percentage (%)	37.8	48.9	8.9	4.4		100

*ASD* atrial septal defect; *AVSD* atrioventricular septal defects; *PDA* patent ductus arteriosus; *TAPVR* total anomalous pulmonary venous return; *TGA* transposition of great arteries; *VSD* ventricular septal defect

^a^Some patients are associated with septal defects

**Fig. 1:**
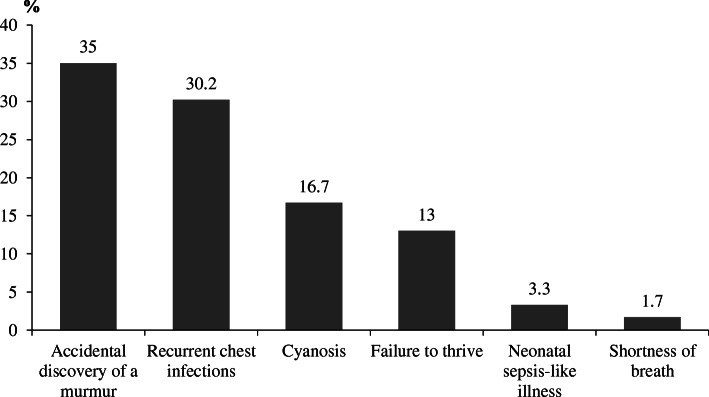
Mode of presentation among studied population

The accidental discovery of a murmur was the most common presenting complaint (Fig. 1). Audible murmurs were detected in 748 (74.4%) and heart failure in 442 (44%) of patients. Hospital admissions were encountered in 469 patients; of them, 270 were admitted due to recurrent chest infections, 91 for cyanotic spells, 75 for failure to thrive, and 33 for neonatal sepsis-like illnesses. Life-saving cardiac interventions were performed in the first year of life for 33 patients; modified Blalock-Tausing shunt for 24 patients with tetralogy of Fallot and balloon atrial septostomy for 9 patients with TGA. Most of the patients (91.3%) were born to young mothers aged < 29 years, and (66.3%) were born to multiparous mothers. Down syndrome was the most common chromosomal anomaly (10.4%) found and atrioventricular septal (AVSD) defect was the most frequently occurring cardiac lesion among them (40.3%). Fetal echocardiography was performed for 47 patients with assisted reproduction; of them, 18 patients had shown abnormal cardiac scanning. Distribution of demographic data and risk factors is shown in Table 2.

**Table 2:** Distribution of demographic data and risk factors

Demographic/risk factor		Number (***n*** = 1005)	Percentage (%)
**Sex**	Male	543	54
	Female	462	46
**Residency**	Urban	790	78.6
	Rural	215	21.4
**Maternal education**	Primary	586	58.3
	Secondary	150	14.9
	Illiterate	269	26.8
**Maternal occupation**	Housewife	922	91.7
	Manual worker	72	7.2
	Desk job	11	1.1
**Paternal occupation**	Farmer	187	18.6
	Manual worker	581	57.8
	Desk job	237	23.6
**Parity**	Nulliparous	339	33.7
	Multiparous	666	66.3
**Maternal age at conception (years)**	≤ 19	427	42.5
	20-29	491	48.8
	30-34	28	2.8
	> 35	59	5.9
**Consanguinity**		448	44.6
**Prematurity**		194	19.3
**Assisted reproduction**		118	11.7
**Family history of CHD**		93	9.2
**Abortions**		71	7.1
**Maternal medical illnesses**	Diabetes	280	27.9
	Bronchial asthma	146	14.5
	Hypertension	74	7.4
	Epilepsy	42	4.2
**Syndromes**	Down S	105	10.4
	Disgorge S	13	1.3
	Others	50	5
**Extracardiac anomalies**	Inguinal hernia	14	1.4
	Diaphragmatic hernia	9	0.9
	Others	13	1.3

Data present in number and percentage

## Discussion

Clinical presentation of CHD is versatile and is age-dependent, and hence, a higher index of suspicion is needed for early diagnosis and treatment [11]. In this study, the commonest presentation for CHD was the accidental discovery (35%) followed by recurrent chest infections (30.2%), cyanosis (16.7%), failure to thrive (13%), neonatal sepsis-like illness (3.3%), and finally shortness of breath (1.7%). In a study done by Otaigbe and Tabansi [3], indications for screening echocardiography were auscultation of a murmur (36%), rapid breathing (19.8%), failure to gain weight (11%), and cyanosis (9.9%), whereas in a study done by George and Frank-Briggs [11], fast breathing and inability to gain weight were the commonest presenting symptoms among CHD children.

Prevalence of murmurs is variable in different studies as it depends on the clinical skills, frequency, and timing of examination as nearly half of newborns with CHD will have no murmurs and possibly no other signs when examined at birth [12]. In this study, we detected audible murmurs in 74.4% of patients. Nearly 2 to 3 out of 1000 neonates with CHD will reveal symptoms during their first year of life. Diagnosis will be made by the first week in 40-50% and by the end of the first month in 50-60% of them [13]. In this study, most of our patients (86.8%) were diagnosed below the age of 1 year (37.8% in the neonatal period, and 49% within the first year of life). Similarly, in a study done by Subramanyan et al. [14], the ages at diagnosis of their CHD cases were in the early infancy and the neonatal periods in 40% and 38% of their studied population respectively.

In this study, we detected a male to female ratio of 1.2:1. This matches with previous studies [3, 15]. Isolated VSD was the most prevalent acyanotic CHD. This matches with most of the previous studies [3, 8, 15], whereas tetralogy of Fallot was the most frequent cyanotic CHD, which is also in agreement with most of the available studies [16–18].

CHD is the most common etiology for the occurrence of heart failure in infancy [19]. In the present study, we detected heart failure in 44% of the studied population. Similarly, Sommers et al. [20] detected heart failure in 39.1% of patients with CHD. The relatively higher prevalence of complications encountered in this study could be attributed to the lack of regular follow up and non-compliance to treatment, which eventually lead to the delay in surgical management.

About 15% of CHD cases could be related to an underlying genetic cause, and a smaller percentage could be attributed to an environmental modifiable risk factor [21]. According to Liu et al. [22], nearly 14% of CHD cases could be prevented by avoidance of exposure to all known risk factors; however, some recent studies detected a higher percentage of about 30% [21, 22]. Through this study, we tried to review the distribution of the perinatal risk factors-already known in literature among our studied population. This work was not designed to study them individually as predictors for the occurrence of CHDs; also, due to missing data in some files; not all the known risk factors were studied. This included maternal body mass index, smoking, anemia, nutritional status, and vitamin and folic acid supplementation during pregnancy.

In this study, the majority of our patients (91.7%) belonged to housewives. Different maternal occupations have been linked to CHDs [23]. Scientific research has not yet confirmed the association between maternal workplace exposure during pregnancy and the possible teratogenic effect on the forthcoming fetus, though there are still some concerns regarding exposure to pesticides, organic solvents, and heavy metals [24].

In Egypt, the prevalence of consanguinity is 29% [25]. The relation between consanguinity and the incidence of CHD had been explored in previous studies [26, 27]. We detected consanguinity and positive family history of CHD in 44.6% and 9.2% of our studied population, respectively. Similarly, Hag et al. [28] detected consanguinity and positive family history in 49% and 14% of their studied population, respectively, whereas Fung et al. [29] detected them in 3.5% and 21.8% respectively and detected 9% prevalence of CHD among first-degree relatives. Also, Nabulsi et al. [5] and AL–Ani [30] detected consanguinity rates of 34.7% and 77.9%, respectively. The differences among various studies reflect the differences in the prevalence of consanguinity among different societies. Moreover, the high-risk factor in closely related parents indicates that consanguinity may act as a genetic predisposition that increases the susceptibility of developing CHD, especially when there is exposure to an environmental risk factor. This highlights the need for public health education regarding the hazards of inbreeding.

In this study, one hundred and eighteen patients (11.7% of our studied population) were the product of assisted reproduction. Children conceived via modern technologies are thought to be at a higher risk for developing birth defects, including CHD [31]. Koivurova et al. [32] detected a fourfold increase in the incidence of CHD among fetuses conceived via in vitro fertilization (IVF). Also, Tararbit et al. [33] found a 40% increase in the risk of CHD among those children.

Chromosomal anomalies account for (8–10%) of syndromatic CHDs [34] with Down syndrome (DS) being the most common chromosomal anomaly seen among them [35]. In this study, syndromatic CHD was present in 16.7% of the studied population (62.5% of them were DS). Fung et al. [29] detected genetic and syndromatic CHD in 9.5% of their studied population. There is geographic variability in the type of the dominant cardiac lesion seen in DS among different countries [36]. In this study, the atrioventricular septal defect was the commonest lesion seen among DS (40.3%). This matches with Benhaourech et al. [37].

The pattern of maternal age as a hazardous factor for congenital defects differs among different countries which imply possible underlying genetic and environmental background rather than only the biological age [38]. Some studies suggested that the gynecological immaturity [39], lack of proper antenatal care, low socioeconomic class, poor diet, and other environmental non-biological factors account for birth defects among young mothers [40]. Other studies had observed the prevalence of CHDs among older mothers [41, 42], whereas Best and Rankin [43] failed to find a strong evidence to support that advanced maternal age is a risk factor for CHD. Older maternal age has been linked to chromosomal-related congenital abnormalities while the risk of maternal age on the non-chromosomal abnormalities is considered negligible [38]. In this study, most of our patients (91.3%) belonged to young mothers (< 29 years old), only 5.9% belonged to mothers older than 35 years at conception, and only 16.7% were associated with syndromatic and chromosomal abnormalities.

Various studies had shown the effect of maternal diabetes as a risk factor for fetal cardiac malformations [44], as well as maternal hypertension, cigarette smoking, and other maternal chronic illnesses [22]. In this study, maternal diabetes, asthma, hypertension, and epilepsy were found in 27.9%, 14.5%, 7.4%, and 4.2% of the studied population, respectively.

Though the exact etiological factors that link the association between increasing maternal parity and the risk of CHD is still unclear, theories include nutrient depletion especially folic acid [45], short inter-pregnancy periods [46], intrauterine exposure to teratogenic viruses (such as rubella) from children sharing the same home environment [47], biological changes in the intrauterine environment and psychological stress in pregnant mothers who are taking care of many children [48]. In this study, most of the patients (66.3%) were born to multiparous mothers, while 33.7% were born to nulliparous mothers. This matches with the meta-analysis done by Yu et al. [49].

In this study, abortions occurred in 7.1% of the studied population. In contrast to Li et al. [50] who failed to find an association between bad obstetric history, recurrent abortions, and the risk of CHD, Abqari et al. [6] detected such an association. Also, Feng et al. [51] found that mothers will have a 24% higher risk of cardiac anomalies in their children if they experienced repeated abortions before. Etiological arguments include possible uterine factors that influence the implanted embryo [52] and associated chronic maternal illnesses [53]. In this study, we also detected prematurity in 19.3% of our studied patients. Tanner et al. [54] found that preterm infants are 2-times prone to CHD when compared to term infants; they detected prematurity in 16% of their CHD patients.

### Study limitations

This work was not designed to study the risk factors as predictors for the occurrence of CHD; instead, it aimed at detecting the frequency of occurrence of those risk factors already documented in literature—among our studied population. Moreover, due to missing data in files, not all the known risk factors were studied, such as maternal body mass index, anemia, nutritional status, antenatal vitamin, and folic acid supplementation. Furthermore, the exact antenatal timing of exposure to teratogen was also missing. In addition, many patients had more than one underlying possible risk factor.

Multi-centric similar studies are needed to be done in different governorates, different geographical areas, Upper and Lower Egypt, rural and urban areas, though we still believe that this study can be considered as a nidus for such studies as it was done in a large University tertiary referral center that receives patients from different geographical areas in Cairo Governorate including those critical, severe, and complicated cases that are neither managed in the Ministry of Health hospitals nor in the private sector.

## Conclusions

This study represents a descriptive epidemiological review of the pattern, clinical spectrum, age of presentation, sex distribution, and risk factor portfolio of CHD among the Egyptian ethnicity. Establishment of a national medical birth registry containing all information about all births in Egypt is needed for adequate surveillance and monitoring of perinatal health problems and congenital birth defects so that preventive measures can be early implemented through interdepartmental collaborations between different medical specialities including obstetricians, pediatricians, and geneticists. Proper and detailed data collection should be fulfilled in the medical records of every single patient.

## Data Availability

All data generated or analyzed during this study are included in this published article.

## Abbreviations

ASD: Atrial septal defect
AVSD: Atrioventricular septal defect
CHD: Congenital heart disease
DS: Down syndrome
HLH: Hypoplastic left heart
PDA: Patent ductus arteriosus
PS: Pulmonary stenosis
TAPVR: Total anomalous pulmonary venous return
TGA: Transposition of great arteries
VSD: Ventricular septal defect
